# The best method for evaluating anteversion of the acetabular component after total hip arthroplasty on plain radiographs

**DOI:** 10.1186/s13018-018-0767-4

**Published:** 2018-04-02

**Authors:** Yang Soo Park, Won Chul Shin, Sang Min Lee, Sang Ho Kwak, Jung Yun Bae, Kuen Tak Suh

**Affiliations:** 1 Department of Orthopedics, Pusan National University Yangsan Hospital, Pusan National University School of Medicine, Yangsan, South Korea; 2 Department of Orthopedic Surgery, Research Institute for Convergence of Biomedical Science and Technology, Pusan National University Yangsan Hospital, Pusan National University School of Medicine, 20 Geumo-ro, Mulgeum-eup, Yangsan, Gyeongsangnam-do 626-770 South Korea

**Keywords:** Anteversion, Acetabular component, Plain radiograph, Total hip arthroplasty

## Abstract

**Background:**

Several radiological methods for measuring the anteversion of the acetabular component after total hip arthroplasty (THA) exist, and no single standardized method has been established. We evaluated the reliability and accuracy of six widely utilized methods (Liaw et al., Lewinnek et al., Widmer, Hassan et al., Ackland et al., and Woo and Morrey) for measuring anteversion on plain radiographs, using a reference standard in the same definition obtained from the PolyWare programme.

**Methods:**

We reviewed 71 patients who underwent primary unilateral THA. The anteversion of the acetabular component was measured on pelvis AP radiographs using five different methods (Liaw et al., Lewinnek et al., Widmer, Hassan et al., and Ackland et al.) and on cross-table lateral radiographs using the method of Woo and Morrey. The values obtained using the PolyWare programme, which determines the anteversion of the acetabular component by edge detection, were regarded as the reference standard.

**Results:**

Intra- and inter-observer reliabilities were excellent for all methods using plain radiographs, including the PolyWare programme. The method of Liaw et al. obtained values similar to those obtained using the PolyWare programme and was thus considered accurate (*P* = 0.447). However, values obtained using the other five methods significantly differed from those obtained using the PolyWare programme and were thus considered less accurate (*P* < 0.001, *P* < 0.001, *P* < 0.001, *P* = 0.007, and *P* < 0.001, respectively).

**Conclusion:**

The method of Liaw et al. is more accurate than other methods using plain radiographs for the measurement of the anteversion of the acetabular component after THA, with reference to the anteversion obtained from the PolyWare programme.

## Background

Malposition of the acetabular component after total hip arthroplasty (THA) is related to dislocation of the prosthetic femoral head, increased polyethylene liner wear, and limited range of motion [1–5]. The orientation of the acetabular component comprises inclination and anteversion. Although the inclination of the acetabular component can be easily measured on plain radiographs, calculation of the anteversion is difficult. There are several radiological methods for measuring the anteversion of the acetabular component after THA, and a single standardized method has not been established. Definition of anteversion differs, depending on various situations. Murray [6] defined three types of anteversion of the acetabular component (anatomical, operative, and radiographic anteversion), each of which are measured on different images (computed tomography (CT), intraoperative assessments, and postoperative plain radiographs). As the anteversion is measured on different axes, depending on the method used, different values can result.

The method described by Woo and Morrey [1] has been widely used to measure the anteversion of the acetabular component on plain radiographs. Although anteversion values vary significantly in serial cross-table lateral views, this method directly evaluates version, without complicated calculations, and allows anteversion and retroversion to be distinguished [7]. In addition, measurements of the anteversion from elliptical projection of the acetabular component on antero-posterior (AP) radiographs have been introduced in numerous reports [1, 2, 8–12]. Previous studies have compared the reliability and accuracy of these measurement methods to that using CT images [13–15]. However, anteversion on CT actually reflects anatomical anteversion, whereas anteversion on plain radiographs represents radiographic anteversion [16]. Therefore, on a theoretical basis, anteversion on CT cannot be used as the reference standard for assessment of radiographic anteversion measurements. Recent studies demonstrate that digital image analysis software using edge detection on plain radiographs, such as the PolyWare programme (Draftware Developers Inc. Vevay, Indiana), accurately measures the anteversion of the acetabular component [17–20]. Unlike those of CT, the results of the PolyWare programme represent radiographic anteversion and thus provide a better reference standard. To our knowledge, no study has evaluated the reliability and accuracy of the widely utilized radiographic anteversion measurement methods using the results from the PolyWare programme as the reference standard.

Therefore, we evaluated the reliability and accuracy of six widely utilized methods for measuring the anteversion of the acetabular component on plain radiographs (Liaw et al. [8], Lewinnek et al. [2], Widmer [9], Hassan et al. [10], Ackland et al. [11], and Woo and Morrey [1]) using the PolyWare programme in the same reference plane as the reference standard.

## Methods

Of the 163 patients who underwent primary THA from December 2012 to February 2014 at our institution, 71 patients who underwent primary unilateral THA were enrolled [21]. We excluded 63 patients with coexisting spinal deformity or previous lumbar intervertebral fusion (to prevent measurement errors based on cross-table lateral radiographs before THA) and 29 patients with low-quality images that were difficult to measure. The patients’ characteristics, including age, sex, body mass index, preoperative diagnosis, the American Society of Anesthesiologists (ASA) classification, and type of prosthesis are shown in Table 1.

**Table 1:** Demographic data of the patients (*n* = 71)

Parameters	Value of number
Mean age at operation (years) (range)	59.4 (24 to 78)
Gender (male/female)	24 / 47
Body mass index (kg/m^2^) (range)	23.5 (19.2 to 28.1)
Preoperative diagnosis (*n*, %)	
Osteoarthritis	31 *(44)*
Femoral head osteonecrosis	27 *(38)*
Femoral neck fracture	7 *(10)*
Rheumatoid arthritis	3 *(4)*
Others	3 *(4)*
ASA score (*n*, %)	
1	28 *(39)*
2	39 *(55)*
3	4 *(6)*
4	0 *(0)*
Type of prosthesis (*n*, %)	
Trilogy cementless cup (Zimmer, Warsaw, IN)	71 *(100)*
Fiber metal taper stem (Zimmer)	71 *(100)*

*ASA* American Society of Anesthesiologists

All surgeries were performed by a single surgeon, who used a posterolateral approach, and included capsuloplasty [22]. All of the procedures were performed using the same type of cementless acetabular (Trilogy, Zimmer, Warsaw, Indiana) and femoral components (Fiber Metal Taper, Zimmer). The mean size of the acetabular component was 50 mm (range 46–60 mm), and two types of prosthetic heads, ceramic or metal, were used.

All radiographs were taken in the same department, using a standardized protocol. Two radiographs were taken for the radiological evaluation: a pelvis AP radiograph and a cross-table lateral radiograph. AP radiographs were taken in the supine position at a source-to-film distance of 115 cm with the X-ray beam centered on the superior aspect of the pubic symphysis and perpendicular to the patient. Cross-table lateral radiographs were performed with the contralateral hip flexed at 90° [9, 23]. The X-ray beam was set parallel to the examination table towards the cephalad at 45° from the patient’s long axis. The film was fixed perpendicular to the examination table using a cassette holder. The anteversion of the acetabular component was measured on the pelvis AP radiograph using the methods of Liaw et al. [8], Lewinnek et al. [2], Widmer [9], Hassan et al. [10], Ackland et al. [11], and PolyWare programme and on the cross-table lateral radiograph using the method of Woo and Morrey [1]. All images were digitally managed and stored through a picture archiving and communication system (Impax PACS, Agfa, Antwerp, Belgium).

### Measurement of the anteversion of the acetabular component on AP plain radiographs

#### The method of Liaw et al. [8]

**Fig. 1:**
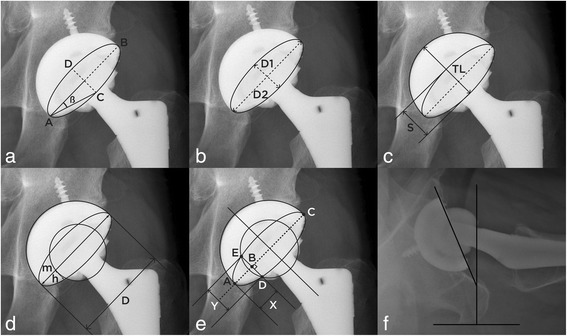
**a** Depiction of the method of Liaw et al. The *β* angle is used to calculate the anteversion. **b** Depiction of the method of Lewinnek et al. The long and short axes of the ellipse are used to calculate the anteversion. **c** Depiction of Widmer’s method. The short axis of the ellipse and total length of the acetabular component are used to calculate the anteversion. **d** Depiction of the method of Hassan et al. The long axis of ellipse and the head edge are used to calculate anteversion. **e** Depiction of the method of Ackland et al. Each perpendicular distance from the end of the ellipse to the cross section between the tangent and the diametrical line is used to calculate the anteversion. **f** Depiction of the method of Woo and Morrey. The cross-table lateral radiograph is used to calculated the anteversion

$$ \mathrm{Version}={\sin}^{-1}\tan\ \beta $$

where *β* angle is the angle between the long axis of the component (AB in Fig. 1a) and the line connecting the end of AB with the end-point of the ellipse (Fig. 1a).

#### The method of Lewinnek et al. [2]

$$ \mathrm{Version}=\arcsin\ \left(D1/D2\right) $$

where D1 is the distance of the short axis of the ellipse and D2 is the long axis of the acetabular component, reflecting the maximum diameter of the implant (Fig. 1b).

#### Widmer’s method [9]

$$ {\displaystyle \begin{array}{l}\mathrm{Version}=\arcsin\ \left(\mathrm{S}\mathrm{hort}\ \mathrm{axis}\ \left(\mathrm{S}\right)/\mathrm{Total}\ \mathrm{length}\ \left(\mathrm{TL}\right)\right)\\ {}=48.05\times \left(\mathrm{S}/\mathrm{TL}\right)-0.3\ \left(\mathrm{if}\ 0.2<\mathrm{S}/\mathrm{TL}<0.6\right)\end{array}} $$

The short axis (S) is the same as D1 in the method of Lewinnek et al. (Fig. 1c). The total length (TL) is an extension of the short axis, reflecting the distance to the apex of the acetabular component. Version is based on the ratio of the short axis to the total length TL (S/TL). The formula is approximately linear when the S/TL value is between 0.2 and 0.6 and can be calculated the second equation above.

#### The method of Hassan et al. [10]

$$ \mathrm{Version}=\arcsin\ \left[\left(h/D\right)/\surd \Big(\right[m/D\left]-\right[{m}^2/{D}^2\left]\Big)\right] $$

where *D* indicates the maximum diameter of the acetabular component; *m* indicates the distance to the point uncovered by the femoral head, which ideally should be the larger value; and *h* indicates the distance of a line perpendicularly drawn from *m* to at the femoral head to the acetabular rim (Fig. 1d).

#### The method of Ackland et al. [11]

$$ \mathrm{Version}=\arcsin\ \left[2\mathrm{y}/2\surd \left(2\mathrm{ax}-{\mathrm{x}}^2\right)\right] $$

where *a* refers to the major axis of the acetabular component (AC in Fig. 1e). An arbitrary tangent is drawn at a right angle to the diameter, and *y* is the distance from the two-cup rims along this tangent (DE in Fig. 1e). Finally, *x* refers to the distance from the end of the ellipse to the cross-section between the tangent and the diametrical line (AB in Fig. 1e).

### Measurement of the anteversion of the acetabular component on lateral plain radiographs

#### The method of Woo and Morrey [1]

Unlike the methods using AP plain radiographs, version is not calculated using a formula; rather, it is directly measured on cross-table lateral radiographs, on which anteversion and retroversion can be distinguished. Version is directly calculated as the angle between the line touching the opening surface of the acetabular component and a line perpendicularly drawn to the table [1, 23] (Fig. 1f).

### Measurement of the anteversion of the acetabular component using the PolyWare programme

**Fig. 2:**
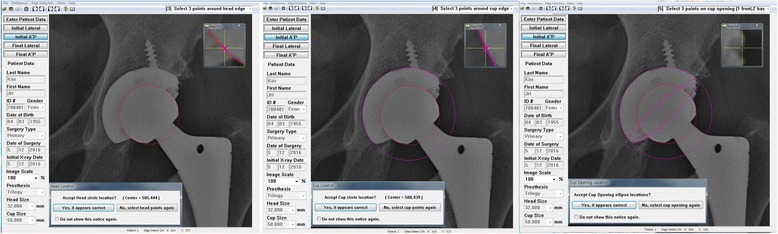
Depiction of the measurement of the anteversion using the PolyWare programme

In the PolyWare programme, the AP plain radiograph is used for edge detection (Fig. 2), with multiple points on the peripheral surface of the acetabular component and prosthetic head. First, three points of the border of the prosthetic femoral head are selected to obtain the best-fitting circle. Secondly, the best-fitting circle of the acetabular component is obtained by the selection of three points of the outer border of the acetabular component. As a next step, three points of the upper and lower edges of the opening of the acetabular ellipse are selected, and the PolyWare programme reconstructs the acetabular component ellipse with a center that overlaps the center of rotation. Finally, the anteversion of the acetabular component is automatically calculated by the PolyWare programme. This value was regarded as the reference standard for the measurement of the anteversion of the acetabular component in the present study.

### Assessment of reliability and accuracy

Reliability can be defined as consistency in measurement. The six methods using plain radiographs were performed independently and at different times by three observers, using the same protocol. The intra-observer reliability for each method was evaluated by one observer and was reassessed twice at intervals of 2 weeks for the images of all 71 patients [24]. The inter-observer reliability across the three observers was calculated for each method. All measurements were calculated with the observer blind to the patient information and the other observers’ values [25]. Accuracy was defined as the proximity to the PolyWare programme value (the reference standard) and was calculated by comparing the values from the six methods using plain radiographs to that obtained using the PolyWare programme.

### Statistical analysis

Intra- and inter-observer reliability were evaluated using intraclass correlation coefficients (ICCs) with 95% confidence intervals (CIs), with one indicating perfect correlation and zero indicating poor correlation. Paired *t* test were used to evaluate the accuracy of each six methods compared to that obtained using the PolyWare programme. Statistical analyses were conducted using SPSS for Windows version 18.0 (SPSS Inc., Chicago, Illinois), and the statistical significance was set at *P* < 0.05.

## Results

The results are summarized in Tables 2 and 3.

**Table 2:** Statistical comparison (reliability)

	PolyWare	Liaw et al. [8]	Lewinneck et al. [2]	Widmer [9]	Hassan et al. [10]	Ackland et al. [11]	Woo and Morrey [1]
ICC for intraobserver reliability (95% CI)	0.962 (0.946 to 0.987	0.933 (0.899 to 0.963)	0.938 (0.891 to 0.963)	0.923 (0.897 to 0.941)	0.953 (0.935 to 0.968)	0.936 (0.916 to 0.958)	0.982 (0.968 to 0.989)
ICC for interobserver reliability (95% CI)	0.948 (0.968 to 0.989)	0.925 (0.889 to 0.953)	0.927 (0.883 to 0.955)	0.954 (0.927 to 0.971)	0.928 (0.905 to 0.941)	0.923 (0.906 to 0.943)	0.981 (0.969 to 0.988)

*ICC* intraclass correlation coefficient, *CI* confidence interval

**Table 3:** Statistical comparison (accuracy)

	PolyWare	Liaw et al. [8]	Lewinneck et al. [2]	Widmer [9]	Hassan et al. [10]	Ackland et al. [11]	Woo and Morrey [1]
Mean anteversion (range)	19.40° (12.60 to 27.80)	19.07° (12.98 to 25.08)	17.19° (10.26 to 24.32)	24.40° (15.61 to 44.79)	17.27° (5.95 to 26.05)	20.83° (9.27 to 33.31)	27.48° (17.20 to 42.40)
*P* value (paired *t* test)		0.447	< 0.001	< 0.001	< 0.001	0.007	< 0.001

### Reliability

The intra- and inter-observer ICCs for measurement of the acetabular component using the PolyWare programme were 0.962 (95% CI 0.946 to 0.987) and 0.948 (95% CI 0.968 to 0.989), respectively (Table 2). All six methods using plain radiographs showed excellent, reproducible measurement of the anteversion of the acetabular component, with high ICC values (ICC > 0.90) indicating excellent intra- and inter-observer reliability. Among the six methods, the method of Woo and Morrey had the highest reproducibility, with an intra-observer ICC of 0.982 (95% CI 0.968 to 0.989) and an inter-observer ICC of 0.981 (95% CI 0.969 to 0.988).

### Accuracy

The overall mean anteversion of the acetabular component using the PolyWare programme was 19.40° (12.6° to 27.8°) (Table 3). The mean value of the anteversion of the acetabular component using the method of Liaw et al. (19.07°, 12.98° to 25.08°) was similar to that using the PolyWare programme (*t* test, *P* = 0.447). In contrast, the measurements using the method of Lewinnek et al., Widmer, Hassan et al., Ackland et al., and Woo and Morrey significantly differ from those obtained using the PolyWare programme (*t* test, *P* < 0.001, *P* < 0.001, *P* < 0.001, *P* = 0.007, and *P* < 0.001, respectively).

## Discussion

We evaluated six radiological methods for measuring the anteversion of the acetabular component after THA on plain radiographs and confirmed that all six methods are consistent and reproducible. However, although the method of Liaw et al. [8] was accurate when compared with the results obtained from the PolyWare programme, the other five methods were less accurate. To the best of our knowledge, the present study is the first investigation of the reliability and accuracy of six widely utilized radiographic anteversion measurement methods using a reference standard appropriately reflecting radiographic anteversion in the same definition and reference plane (the results of the PolyWare programme).

Proper positioning of the acetabular component is critical to successful THA [4, 5, 26]. Although the ideal anteversion value remains controversial, with Charnley et al. [27] stating that there is no absolute ideal value, most studies recommend an anteversion value between 5° and 30° [2, 28]. Revision operation should be a strong consideration in patients who have a dislocation with obvious malposition of the acetabular component after THA [2, 26]. Furthermore, accurate assessment of the acetabular component position at the time of revision surgery is crucial in preventing dislocation after the revision surgery. In general, the measurement of acetabular anteversion preoperatively could be confined to CT scans or cross-table lateral radiographs; however, various measurement methods can be used postoperatively because of the presence of the acetabular component. However, there is currently no single standardized method, and which method is the most accurate is unclear. In general, the most desirable method would be readily available, consistent, reproducible, economical, quick, and easy to interpret. Plain radiographs remain the most universally available and economical method for the postoperative measurement of the component’s position.

The reliability and accuracy among various radiological methods have not been studied in depth, and previous studies show conflicting results [14, 15, 29–32]. Inconsistent definitions and reference planes for the anteversion of the acetabular component interfere with the comparison of reported methods. Several studies have compared various radiological methods for measuring anteversion of the acetabular component using the CT results as a reference standard (Table 4) [14, 15, 29]. For example, Marx et al. [14] compared the accuracy of five plain radiographic methods (McLaren [3], Ackland et al. [11], Pradhan [33], Widmer [9], and Hassan et al. [10]), using the CT measurement as a reference standard, and concluded that Widmer’s method had only small errors and could be recommended for determining anteversion based on plain radiography. Similarly, Nho et al. [15] compared the accuracy of six plain radiographic methods (Lewinnek et al. [2], Widmer [9], Hassan et al. [10], Ackland et al. [11], Liaw et al. [8], and Woo and Morrey [1]), using the CT measurement as a reference standard, and reported that the methods of Lewinnek et al., Hassan et al., Liaw et al., and Woo and Morrey were accurate. Nomura et al. [29] compared the accuracy of five plain radiographic methods (Lewinnek et al. [2], Widmer [9], Liaw et al. [8], Pradhan [33], and Woo and Morrey [1]), using the CT measurement as a reference standard, and reported that Widmer’s method was the best at evaluating the anteversion of the acetabular component on plain radiographs. However, as defined by Murray [6], CT anteversion reflects anatomical anteversion while plain radiographs reflect radiographic anteversion. In general, anatomical anteversion and operative anteversion are always greater than radiographic anteversion [29]. In theory, a comparison between CT anteversion and that obtained using methods based on plain radiographs is inappropriate, as the reference planes in anatomical anteversion and radiographic anteversion are different. Nomura et al. [29] suggested that the anteversion measured on pelvic CT images resliced parallel to a functional coronal plane reflects the radiographic anteversion; however, this has not yet been clearly demonstrated. In addition, the CT scan exposes patients to a greater dose of radiation and is too expensive and time-consuming for routine use.

**Table 4:** Reported accuracy with measurements for anteversion

Study	Methods	Anteversion (SD or range)	*P* value (*t* test)	Intraobserver reliability ICC	Interobserver reliability ICC
Marx et al. [14]	CT	29.9° (8.7)			
	McLaren [3]	15.4° (7.7)	< 0.0001		
	Ackland et al. [11]	15.6° (8.2)	< 0.0001		
	Pradhan [33]	15.4° (8.4)	< 0.0001		
	Widmer [9]	23.5° (10.5)	< 0.0001		
	Hassan et al. [10]	15.5° (8.3)	< 0.0001		
Nho et al. [15]	CT	26.80° (7.85)		0.954	0.982
	Lewinnek et al. [2]	26.90° (6.19)	0.901	0.938	0.943
	Widmer [9]	19.14° (3.32)	< 0.001	0.920	0.961
	Hassan et al. [10]	26.11° (5.26)	0.425	0.893	0.936
	Ackland et al. [11]	15.66° (3.53)	< 0.001	0.914	0.865
	Liaw et al. [8]	28.48° (6.25)	0.058	0.915	0.929
	Woo and Morrey [1]	28.08° (6.27)	0.171	0.993	0.955
Nomura et al. [29]	CT	23.8° (8.6 to 38.7)		0.971	0.975
	Lewinnek et al. [2]	19.2° (7.3 to 32.8)	< 0.001	0.930	0.946
	Widmer [9]	22.9° (10.1 to 40.4)	0.088	0.925	0.944
	Liaw et al. [8]	19.7° (7.6 to 33.9)	< 0.001	0.922	0.929
	Pradhan [33]	18.5° (6.6 to 37.0)	< 0.001	0.913	0.923
	Woo and Morrey [1]	27.7° (8.8 to 43.7)	< 0.001	0.976	0.981

*SD* standard deviation, *ICC* intraclass correlation coefficient

The usefulness of the PolyWare programme in THA has previously been reported [18–20]. The PolyWare programme has been used practically to accurately measure the wear of polyethylene liners in THA [34, 35]. The precise orientation of the acetabular component must be determined to accurately measure the wear of the polyethylene liners. A best-fit ellipse of the acetabular component is created from landmarks collected by the user, and a three-dimensional stereoscopic image is automatically reconstructed from two-dimensional planar images of plain radiographs. This allows the measurement of the anteversion of the acetabular component, which has a three-dimensional structure in three-dimensional space. The results after processing reflect radiographic anteversion. In addition, the PolyWare programme allows the user to input pelvic landmarks to accommodate a change in the position of the pelvis. Furthermore, the PolyWare programme does not require additional radiographs, so there is no radiation exposure. Measurements of the anteversion using the PolyWare programme were regarded as the reference standard in the present study.

There are few reports regarding differences among various methods for measuring the anteversion of the acetabular component on plain radiographs using the same radiographic definition. We evaluated the reliability of six methods for assessing radiographic anteversion and found that all six methods showed excellent, reproducible measurements of the anteversion of the acetabular component, with high ICC values. The mean acetabular anteversion using the PolyWare programme was 19.40° in the present study. In addition, we evaluated the accuracy of these six methods using the results of the PolyWare programme, with the same definition of anteversion, as the reference standard. Based on the present results, the method of Liaw et al. [8] is accurate. Although the other methods were less accurate, the method of Woo and Morrey [1] showed the highest intra- and inter-observer reliability.

The present study has several limitations. First, the study was retrospective in nature. Secondly, if ceramic or metal liners are used (unlike in the present study), the shades of the acetabular components will not show distinctly because the border of the ellipse is difficult to identify, making anteversion measurement difficult. Thirdly, patient positioning during the performance of the radiographs could influence the measurement of the anteversion. However, when radiographic images are obtained using standardized patient positions, the variability in measurements is minimized. Finally, the present study cannot explain why the method of Liaw et al. [8] is accurate; we presume that the accuracies are different due to differences in the mathematical formulae for calculating anteversion. Furthermore, the method of Liaw et al. [8] is well established.

## Conclusion

In conclusion, we found that the method of Liaw et al. [8] using AP radiographs appears to be more accurate than other methods for measuring the anteversion of the acetabular component after THA on plain radiographs, using the anteversion obtained from the PolyWare programme as the reference standard. As the use of different definitions and reference planes interferes with the comparison of various methods, future studies should be designed using a consistent definition for the anteversion of the acetabular component.

## Abbreviations

AP: Antero-posterior
ASA: American Society of Anesthesiologists
CI: Confidence interval
CT: Computed tomography
ICC: Intraclass correlation coefficient
THA: Total hip arthroplasty
